# The protective role of small heat shock proteins in cardiac diseases: key role in atrial fibrillation

**DOI:** 10.1007/s12192-017-0799-4

**Published:** 2017-05-08

**Authors:** Xu Hu, Denise M. S. Van Marion, Marit Wiersma, Deli Zhang, Bianca J. J. M. Brundel

**Affiliations:** Department of Physiology, Institute for Cardiovascular Research, VU University Medical Center, De Boelelaan 1117, 1081 HV Amsterdam, The Netherlands

**Keywords:** Heat shock protein, Atrial fibrillation, Small HSP (HSPB), Proteostasis

## Abstract

Atrial fibrillation (AF) is the most common tachyarrhythmia which is associated with increased morbidity and mortality. AF usually progresses from a self-terminating paroxysmal to persistent disease. It has been recognized that AF progression is driven by structural remodeling of cardiomyocytes, which results in electrical and contractile dysfunction of the atria. We recently showed that structural remodeling is rooted in derailment of proteostasis, i.e., homeostasis of protein production, function, and degradation. Since heat shock proteins (HSPs) play an important role in maintaining a healthy proteostasis, the role of HSPs was investigated in AF. It was found that especially small heat shock protein (HSPB) levels get exhausted in atrial tissue of patients with persistent AF and that genetic or pharmacological induction of HSPB protects against cardiomyocyte remodeling in experimental models for AF. In this review, we provide an overview of HSPBs as a potential therapeutic target for normalizing proteostasis and suppressing the substrates for AF progression in experimental and clinical AF and discuss HSP activators as a promising therapy to prevent AF onset and progression.

## AF progression by structural remodeling

Atrial fibrillation (AF) is an age-related tachyarrhythmia in both left and right atria, which can be caused by underlying (heart) conditions, such as valvular heart disease, congestive heart disease, ischemic cardiomyopathy, obesity, diabetes mellitus, and hypertension (Dobrev et al. 2012; Hoogstra-Berends et al. 2012). The goal of AF therapy is, ideally, to abolish AF episodes and to restore normal sinus rhythm. Unfortunately, treatment of AF remains difficult, which is caused by the persistent and progressive nature of this arrhythmia. There are strong indications that remodeling of the structure of atrial cardiomyocytes underlies electrophysiological and contractile dysfunction and AF perpetuation (de Groot et al. 2010). Structural remodeling includes degradation of sarcomeres (the smallest contractile units of the cardiomyocytes), namely myolysis, by proteases such as calpain (Brundel et al. 2002; Ke et al. 2008) and disruption of the microtubule network (Zhang et al. 2014), which result in impaired electrical coupling and functional recovery to sinus rhythm after pharmacological and electrical cardioversion (Ausma et al. 1997; Kirubakaran et al. 2015; Todd et al. 2004). Importantly, structural changes are already presented when a patient enters the clinic, for the first time, with an episode of AF. Since the current available therapy is directed at alleviation of electrophysiological changes (rhythm control), it has limited effect on patient’s outcome. Therapeutic approaches that counteract the pathways conveying AF-induced structural remodeling may offer superior therapeutic perspectives. Recent research findings indicate that derailment of proteostasis, i.e., the homeostasis of protein production, function, and degradation, constitutes an important factor for the induction and progression of AF. In addition, it was observed that especially small heat shock proteins (HSPBs) convey protective effects against derailment of proteostasis and thereby attenuate structural remodeling, AF onset, and progression.

## Proteostasis and role for HSPs

It has been recognized that a proper function of cells and organisms depends critically on the maintenance of a healthy proteostasis (Balch et al. 2008; Kampinga and Bergink 2016). Proteostasis is particularly important in long-lived post-mitotic cardiomyocytes, since they display limited regenerative capacity. Proteostasis involves controlling the concentration, conformation, binding interaction, kinetics, and location of individual proteins. Derailment of cellular proteostasis results in many systemic diseases, including cardiovascular disorders (Balch et al. 2008). Cells respond to a loss of proteostatic control by inducing a heat shock response (HSR), upon which HSPs are expressed. Interestingly, in the heart, numerous HSPs are already expressed at high levels, especially the family of small HSP members: HSPB1, HSPB5, HSPB6, HSPB7, and HSPB8. These HSPBs are considered to constitute the cell’s first line of defense against proteostasis derailment by stabilizing the sarcomere (Brundel et al. 2006a; Ke et al. 2008). In general, HSPs act as molecular chaperones to facilitate protein folding, localization, degradation, and function, thereby maintaining proteostasis and preventing various forms of cardiomyocyte damage (Westerheide and Morimoto 2005). Indeed, HSPs were found to play a protective role in various cardiovascular diseases, including AF. Two studies reported induced expression of mitochondrial HSPs, HSPD1, HSPE1, and mortalin (HSPA9B) in atrial tissue of patients with AF. These HSPs may play a protective role by maintaining mitochondrial integrity and capacity for ATP generation (Kirmanoglou et al. 2004; Schafler et al. 2002). Unfortunately, no mechanistic studies have been performed to conclusively address their function. Other studies revealed induced HSPA1A expression in atrial tissue of patients undergoing cardiac surgery. Higher HSPA1A expression correlated with lower incidence of post-surgery AF, suggesting a cardioprotective role for HSPA1A (Mandal et al. 2005; St Rammos et al. 2002). A key role for HSPB members in the protection against AF onset and progression was identified by several studies (Brundel et al. 2006a, b; Ke et al. 2011; Zhang et al. 2011). Interestingly, it was found that overexpression of HSPB1 protects against contractile dysfunction by conservation of the cardiomyocyte structure in the tachypaced HL-1 cardiomyocyte model for AF and in clinical AF (Brundel et al. 2006a), suggesting HSPB1 to represent a druggable target in AF.

## Key role for HSPB members in the prevention of cardiac diseases

### HSPB members

The family of HSPBs consists of at least ten members, and they are expressed in various human tissues (Vos et al. 2009) (Table 1). HSPB members are defined by a conserved C-terminal domain of approximately 90 amino acids (the α-crystallin domain) flanked by a variable length N-terminal arm and a more conserved C-terminal extension (Bakthisaran et al. 2015). Some HSPB members, including HSPB1, HSPB5, and HSPB8, are thought to assemble into homo- and/or heterogeneous oligomeric complexes, which dissociate into smaller multimers upon stress. Another important characteristic is that various HSPB members can be phosphorylated, which changes their activity and oligomeric state (Vos et al. 2008).

**Table 1:** Characteristics of HSPB members

Gene name	Protein name	Alternative name	Molecular weight (kDa)	Expression in heart	References	Other tissue expressions
HSPB1	HSPB1	DmHSP23, HSP25, HSP27	22.783	+	Golenhofen et al. (2004) Vos et al. (2009)	Uterus, skin, platelets, brain, kidney, some tumor cells
HSPB2	HSPB2	MKBP	20.233	+	Sugiyama et al. (2000) Vos et al. (2009) Ishiwata et al. (2012)	Skeletal muscle
HSPB3	HSPB3	HSPL27	16.966	−	–	Skeletal muscle
HSPB4	HSPB4	αA-Crystallin, CRYAA, CRYA1	19.909	−	–	Lens of eye, spleen
HSPB5	HSPB5	αB-Crystallin, CRYAB, CRYA2	20.159	+++	Bennardini et al. (1992) Vos et al. (2009) Cubedo et al. (2016)	Lens of eye, vascular wall muscle, lung, kidney, brain, some tumor cells
HSPB6	HSPB6	HSP20	17.136	++	Verschuure et al. (2003) Golenhofen et al. (2004) Fan et al. (2015) Vos et al. (2009) Fan and Kranias (2011)	Skeletal muscle, stomach, liver, lung, kidney, platelet
HSPB7	HSPB7	CvHSP	18.611	++++	Krief et al. (1999) Verschuure et al. (2003) Golenhofen et al. (2004) Vos et al. (2009)	Skeletal muscle
HSPB8	HSPB8	HSP22, H11 kinase (H11K)	21.604	+++	Verschuure et al. (2003) Vos et al. (2009)	Skeletal muscle, stomach, liver, lung, kidney, brain
HSPB9	HSPB9	FLJ27437	17.486	−	–	Testis
HSPB10	HSPB10	ODF1	28.366	−	–	Testis

HSPBs have at least three, not mutually exclusive, biochemical functions within the proteostasis regulation. Firstly, both in vitro and in vivo findings suggested that some HSPB members act as ATP-independent chaperones by preventing irreversible protein aggregation (Carra et al. 2009; Chowdary et al. 2004; Sanbe et al. 2007). Oligomeric dynamics seem to be crucial for such HSPB activities (van Montfort et al. 2001). HSPB clients may also be processed (renaturation or degradation) through cooperation with ATP-dependent chaperones (Mogk et al. 2003; Veinger et al. 1998). As such, stress-induced protein damage, which may also occur in AF, can be prevented. Secondly, several HSPB members are associated with cytoskeletal proteins in a phosphorylation-dependent manner (Golenhofen et al. 2004; Landry and Huot 1995; Lavoie et al. 1995). This results in stabilization of cytoskeletal structures and increased resistance to stress situations, including AF. Finally, HSPB members are found to inhibit the activation of proteases and as such may prevent the activation of calpain, which was found to become activated in clinical AF (Brundel et al. 2002; Zhang et al. 2011).

### Relevant HSPB family members for heart function: functional similarities and divergence

Various HSPB members are expressed at high levels in the heart (Golenhofen et al. 2004; Verschuure et al. 2003) (Table 1). One of these members is HSPB1. HSPB1 can exist as high or low molecular weight structures. Under normal, non-stressed conditions, a high molecular weight structure is the most predominant form. During proteotoxic stress, its level decreases whereas the level of phosphorylated low molecular weight structures increases (Vos et al. 2008). In addition to the role of HSPB1 in assisting in refolding and/or targeting denatured proteins, another well-studied role of HSPB1 is its ability to interact with several cytoskeletal proteins, including actin, intermediate filaments, and microtubules (Landry and Huot 1995; Vicart et al. 1998). In heart tissue, HSPB1 is found associated with sarcomeres and thereby was found to be cardioprotective (Brundel et al. 2006a).

HSPB2 associates specifically with dystrophy myotonic protein kinase (DMPK) and therefore is called a DMPK-binding protein, indicating its importance in muscle maintenance (Kadono et al. 2006; Suzuki et al. 1998). It is highly expressed in heart and skeletal muscle and was found to have protective effects against cardiac diseases, such as cardiac hypertrophy and ischemia heart diseases (Ishiwata et al. 2012; Nakagawa et al. 2001; Sugiyama et al. 2000). Also, HSPB2 was found to be associated with the outer membrane of mitochondria, thereby regulating the mitochondria permeability transition and calcium uptake in mitochondria. Overexpression of HSPB2 was found to conserve ATP synthesis in mice with ischemic/reperfusion injury (Nakagawa et al. 2001). Mice with specific knockout of HSPB2 show, upon ischemic stress, reduced mitochondria respiration rates and ATP production as well as suppression in expression of several metabolic and mitochondrial regulators (Ishiwata et al. 2012). These findings imply that HSPB2 is cardioprotective via maintenance of mitochondrial function and metabolic activity during cardiac stress. This role has been confirmed in a study utilizing a double knockout of HSPB2 and HSPB5. Here, inhibition of mitochondrial calcium signaling and, consequently, a reduction in ATP synthesis were observed during ischemia/reperfusion (Kadono et al. 2006). Findings from the study of Golenhofen et al. imply that the increased calcium in the cytosol, due to knockout of HSPB2, may modify the calcium sensitivity of myofibrils, contributing to malfunction of cardiac contractility (Golenhofen et al. 2006). Interestingly, mice overexpressing cardiac HSPB2 revealed lower levels of cardiac biomarker troponin I in the blood after ischemia/reperfusion injury, indicating that troponin I levels in heart tissue are conserved, thereby preserving contractile function of the heart (Grose et al. 2015).

HSPB3 and HSPB4 are not expressed in the heart (Vos et al. 2009), whereas HSPB5 co-localizes on the I-band and M-line region of sarcomeres in cardiomyocytes (van de Klundert et al. 1998). HSPB5 is known to bind and stabilize intermediate filaments, actin microfilaments, and sarcomeric proteins, including actin, desmin, and titin (Bullard et al. 2004; Ghosh et al. 2007; Perng et al. 1999). Like HSPB1, HSPB5 also plays an important role in stabilization of the cytoskeleton as it is expressed together with HSPB1 to associate with sarcomeric proteins (Vicart et al. 1998). Mutations in HSPB5 are associated with a broad variety of neurological, cardiac, and muscular disorders. The R120G mutation results in an irregular protein structure and defective chaperone-like function (Bova et al. 1999), which may accelerate the accumulation of desmin aggregation, thereby leading to desmin-related myopathy and also early onset of cardiomyopathy (Selcen and Engel 2003; Vicart et al. 1998).

HSPB6 is abundantly expressed in skeletal muscle and heart in two complex formations: 43 kDa dimers and 470 kDa multimers. HSPB6 binds to itself and other HSPBs (HBPB1, HSPB5, and HSPB8) (Pipkin et al. 2003). Recently, HSPB6 overexpression was found to result in enhanced cardiac function by interacting with protein phosphatase 1, thereby inducing Ca^2+^ cycling and sarcoplasmic reticulum Ca^2+^ load (Qian et al. 2011). In addition, in HSPB6 transgenic rat ventricular cardiomyocytes, HSPB6 increases the phosphorylation at specific sites of the calcium regulatory protein phospholamban, via inhibition of protein phosphatase 1. As such, HSPB6 promotes the Ca^2+^ cycling in the sarcoplasmic reticulum and enhances the contractile function of the cardiomyocyte (Qian et al. 2011). Moreover, another study described HSPB6 to reduce the myocardial infarcted area, thereby conserving the heart integrity in mice with ischemia/reperfusion injury (Fan et al. 2006). Besides, the phosphorylation of HSPB6 at serine 16 was found to be required for attenuating ischemia/reperfusion-induced cell injury in mice, as the non-phosphorylatable HSPB6 induced apoptosis and necrosis, suppressed the autophagy activity, and subsequently depressed the cardiac functional recovery during ischemia and reperfusion (Qian et al. 2009).

HSPB7 is expressed in heart and skeletal muscle. In aged muscle, it was shown that both HSPB5 and HSPB7 expressions are dramatically increased (Doran et al. 2007). HSPB7 upregulation is also found in the muscular dystrophy-affected diaphragm, indicating that HSPB7 levels are induced under stress conditions. Furthermore, HSPB7 protects cells from protein aggregation, likely by facilitating cargo delivery to autophagosomes (Vos et al. 2010). Interestingly, HSPB4, HSPB6, or HSPB7 could not enhance the cellular capacity to chaperone heat-denatured luciferase, in contrast to HSPB1, indicating further functional differentiation of the HSPB members (Vos et al. 2010, 2011). In addition, co-localization of HSPB7 on myofibrils in cardiomyocytes is observed (Golenhofen et al. 2004), suggesting a protective role via conservation of the sarcomeric structure.

HSPB8 is strongly expressed in striated and smooth muscles, brain, and keratinocytes (Vos et al. 2008). Like HSPB1 and HSPB5, HSPB8 can also be phosphorylated in vitro. In contrast to HPB1 and HSPB5, phosphorylation of HSPB8 only marginally affects its tertiary and quaternary structure. Both wild-type and phosphorylated HSPB8 exist as low molecular mass oligomers. Unlike HSPB1 and HSPB5, where phosphorylation increases chaperone activity and reduces oligomeric size, phosphorylation of HSPB8 results in larger oligomeric structures and severely lowered chaperone activity (Basha et al. 2006). In in vitro experiments, HSPB8 interacts with several proteins and forms stoichiometric complexes with Bag3, a co-factor of HSPA1A (Carra et al. 2008a). The Bag3/HSPB8 complex was found to induce both translational arrest and autophagy, which may be beneficial in response to irreparable protein damage (Carra et al. 2008b, 2009). In addition, HSPB8 is cardioprotective in experimental models of myocardial ischemia. Overexpression of HSPB8 promotes cardiomyocyte survival after ischemia in mice (Depre et al. 2006) and attenuates the myocardial damage and contractile dysfunction in pig (Chen et al. 2011), whereas depletion of HSPB8 in mice with pressure overload contributes to the cardiac dysfunction and accelerates transition to heart failure (Qiu et al. 2011). Furthermore, studies show HSPB8 to conserve mitochondrial function and energy production, thereby attenuating oxidative stress in infarcted hearts (Marunouchi et al. 2014). In contrast to these beneficial effects of HSPB8 on cardiomyocyte function, overexpression of HSPB8 was also found to induce cardiac hypertrophy both in in vitro and in vivo model systems and reexpression of the cardiac fetal gene program and provoked cell growth pathways as well as proteasome activities (Depre et al. 2002; Hedhli et al. 2008). Therefore, the function of HSPB8 seems two-edged in heart diseases: HSPB8 reveals beneficial effects on myocardial ischemia by conserving the mitochondrial function and energy production, and HSPB8 is a mediator of cardiac hypertrophy and thereby results in heart failure.

Interestingly, various HSPB family members have common functions by translocating from cytoplasm to specific sarcomeric proteins upon different forms of stress (Table 2). During aging, HSPB1 was found to translocate from the cytoplasm of ventricular cardiomyocytes to sarcomeric actin in the Z line (Lutsch et al. 1997). Under acidic stress, HSPB1 translocates from the cytosol to the unfolded Ig domain of Titin on the I-band, to prevent its aggregation resulting in maintenance of titin function. Moreover, HSPB1 is co-localized with the titin spring in the elastic I-band region in dialated cardiomyopathy patients, while HSPB1 is mainly expressed in cytoplasm of cardiomyocytes in the healthy heart. In addition, phosphomimicking HSPB1 mutants did not alter its binding affinity to titin, compared to the wild-type HSPB1 (Kotter et al. 2014), indicating that binding of HSPB1 to titin is phosphorylation dependent. In the ischemic heart, co-localization of HSPB2 at the Z line of sarcomeres was enhanced (Yoshida et al. 1999). Moreover, in in vivo studies, HSPB5 was found to be soluble in the cytosol of cardiomyocytes under normal control conditions, while in the ischemic heart, HSPB5 was phosphorylated resulting in the transition of soluble HSPB5 to insoluble fractions and translocation from the cytosol to myofibrils. Furthermore, it was demonstrated in a pig model that HSPB5 strongly binds to titin after translocation to myofibrils. The chaperone activity is required to prevent the unfolding and irreversible derailment of myofibrils (Golenhofen et al. 1998, 1999, 2002). HSPB6 associates with HSPB5 and localizes in the distinct transverse bands in the similar pattern as sarcomeric actin, indicating that it probably modulates the contractile dynamics in cardiac myocytes through associating with sarcomeric actin (Pipkin et al. 2003).

**Table 2:** HSPB binding on sarcomere structural proteins

Protein name	Experimental model	Myofibrillar protein targets	Phosphorylation dependency	Stress conditions	Reference
HSPB1	Ventricular cardiomyocytes	Sarcomeric actin	–	Aging	Lutsch et al. (1997)
	Cardiomyocytes	Titin	Independent	Acidic stress; dialated cardiomyopathy	Kotter et al. (2014)
	Soleus muscle fibers	Z disc	Dependent	Hindlimb reloading	Kawano et al. (2012)
	Biceps brachii muscle	Z disc	–	High-force eccentric exercise	Paulsen et al. (2009)
HSPB2	Cardiomyocytes	Z line	–	Ischemia	Yoshida et al. (1999)
HSPB5	Cardiomyocytes	Titin	Dependent	Ischemia	Golenhofen et al. (1998, 1999, 2002)
HSPB6	Cardiomyocytes	Sarcomeric actin	–	Normal condition	Pipkin et al. (2003)
HSPB7	Skeletal muscle	Dimerized filamin C on Z line	–	HSPB7 KO-induced myopathy	Juo et al. (2016)
HSPB8	–	–	–	–	–

HSPB3 and HSPB4 are not expressed in heart; − is unknown; KO is knockout

## Protective role of HSPB members in atrial fibrillation

So far, various HSPB members are found to be protective against AF (Table 3). In atrial tissue of patients with AF, HSPB1 localizes at sarcomeres (Brundel et al. 2006a). Furthermore, HSPB1 overexpression prevents the degradation of sarcomeric proteins in tachypaced HL-1 cardiomyocytes (Brundel et al. 2006a), indicating a prominent role for HSPB1 in conservation of the sarcomeric structure and function. Next to HSPB1, also HSPB6, HSPB7, and HSPB8 display protective effects against cardiomyocyte remodeling in tachypaced HL-1 cardiomyocytes (Ke et al. 2011). As several HSPB members can form hetero-oligomeric complexes with each other, the protective effect of the various members may be due to the supportive oligomeric structures with HSPB1 (van Montfort et al. 2001; Vos et al. 2008). As downregulation of endogenous HSPB1 did not impair the protective effects of HSPB6, HSPB7, and HSPB8 in tachypaced HL-1 cardiomyocytes, their effects seem independent of endogenous HSPB1 (Ke et al. 2011). Interestingly, all the protective HSPB members were able to reduce the formation of F-actin stress fibers, supporting the view that actin is the key target of the HSPB members in AF. Yet, the mode of action in preventing F-actin stress bundle formation of the four protective HSPB members seems to differ. Whereas HSPB8 interferes with the upstream tachypacing-induced RhoA GTPase activation, HSPB1, HSPB6, and HSPB7 do not. Rather, HSPB1, HSPB6, and HSPB7 bind to actin and directly inhibit G- to F-actin polymerization and/or stimulate depolymerization, indicating a protective role against tachycardia remodeling downstream of RhoA GTPase activation (Ke et al. 2011).

**Table 3:** Summary of roles of several HSPBs in atrial fibrillation

HSPB	OE	Experimental model	Consequences	References	Other studied cardiac diseases
HSPB1	+	HL-1 cardiomyocytes	↑ CaT; ↑ CS; ↓ myolysis Co-localization with myofibrils and actin filament	Brundel et al. (2006a) Brundel et al. (2006b) Ke et al. (2011)	Ischemia/reperfusion (Vander Heide 2002) (Lu et al. 2008) Heart failure (Liu et al. 2007)
		*Drosophila*	↑ Heart contraction rate ↓ Duration of arrhythmia episode ↑ Heart wall shortening ↓ Ultrastructural damage ↓ Calpain activity	Zhang et al. (2011)	
HSPB6	+	HL-1 cardiomyocytes	↑ CaT ↓ F-actin stress fiber	Ke et al. (2011)	Ischemia/reperfusion (Qian et al. 2011) (Nicolaou et al. 2008) (Islamovic et al. 2007)
HSPB7	+	HL-1 cardiomyocytes	↑ CaT ↓ F-actin stress fiber	Ke et al. (2011)	–
HSPB8	+	HL-1 cardiomyocytes	↑ CaT ↓ RhoA GTPase activity ↓ F-actin stress fiber	Ke et al. (2011)	Ischemia/reperfusion (Depre et al. 2006) Hypertrophy (Chen et al. 2011) (Hedhli et al. 2008)

*HL-1 cardiomyocytes* mouse atrial cardiomyocytes, *CaT* calcium transient, *CS* cell shortening, *OE* overexpression

Next to the protective effects on F-actin stress bundle formation, HSPB1 conserves the calcium handling. HSPB1 overexpression protects against loss in Ca^2+^ transients and cell shortening in tachypaced HL-1 cardiomyocytes and this protective effect is phosphorylation-dependent, as a non-phosphorylatable HSPB1 mutant did not show an effect (Brundel et al. 2006a). In addition, the protective effect on the calcium handling may involve the direct modulation of ion channel function or modulation of specific kinases, resulting in the conservation of ion currents, including the L-type Ca^2+^ current (Christ et al. 2004). Previously, HSPs were found to regulate ion channel function in the heart and brain (Armstead and Hecker 2005; Ficker et al. 2003; Kashlan et al. 2007; Krieger et al. 2006). Some HSPs were found to interact directly with ion channels, such as HSPB5 with Na^+^ channels (Kashlan et al. 2007) and HSPA1A with cardiac K^+^ channel HERG (Ficker et al. 2003) and voltage-gated Ca^2+^ channels (Krieger et al. 2006), suggesting a possible role for HSPBs in AF attenuation by interacting with ion channels.

HSPBs may also protect against AF by affecting signaling cascades that are activated by AF. HSPB1 associates with several kinases, such as IkappaB kinase and c-Jun N-terminal kinase (JNK), thereby suppressing activation of the transcription factor NF-κB (Kammanadiminti and Chadee 2006; Park et al. 2003). Interestingly, these kinases were reported to be modulated during AF (Cardin et al. 2003; Li et al. 2001; Qi et al. 2008).

**Fig. 1:**
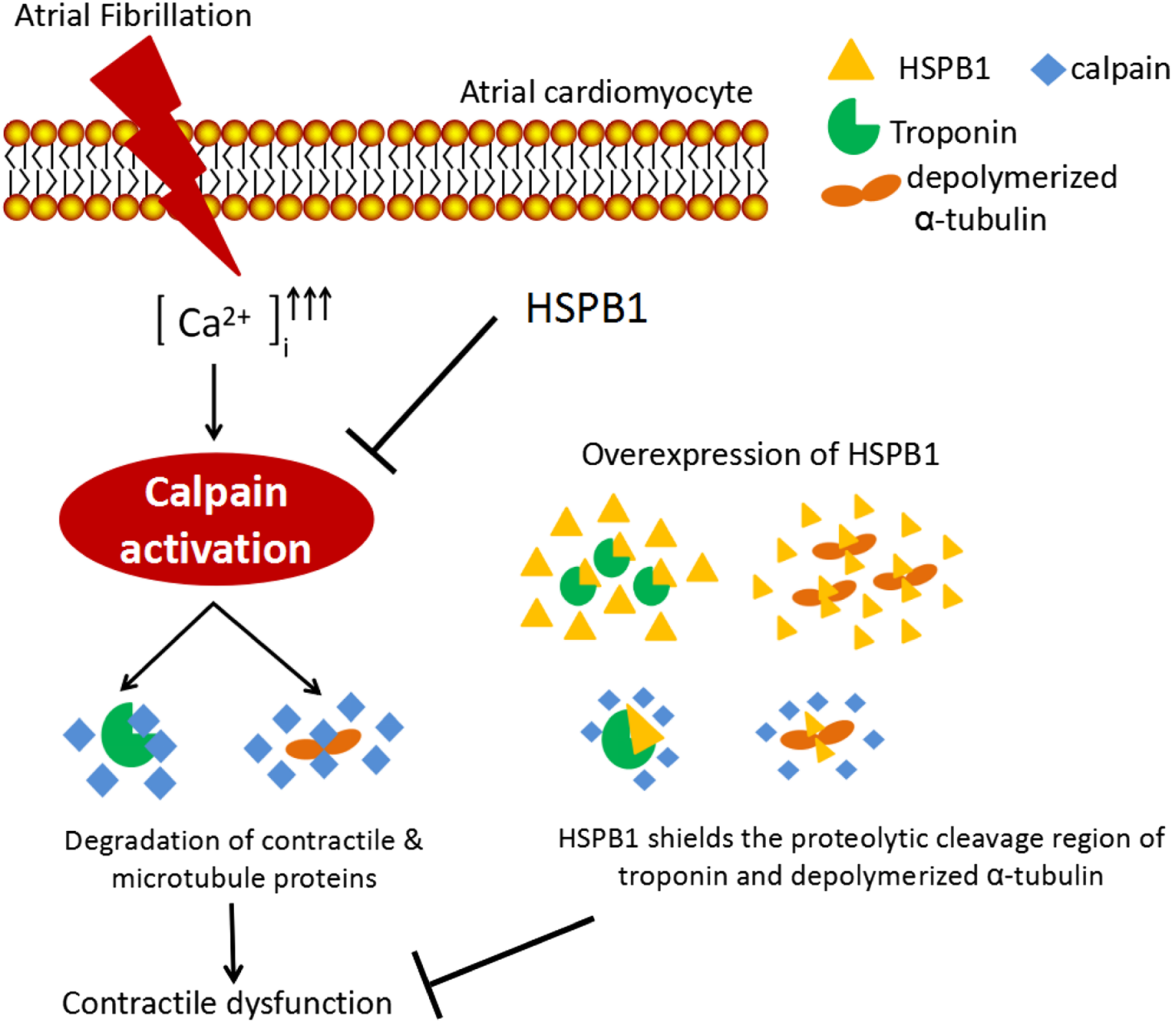
AF induces a calcium overload in cardiomyocytes, which activates calcium-dependent neutral protease calpain. Calpain degrades contractile proteins and microtubule network resulting in structural remodeling, contractile dysfunction of cardiomyocytes, and AF progression. Elevated HSPB1 is found to inhibit calpain activity in tachypaced *Drosophila*. In addition, HSPB1 prevents degradation of cardiac troponins and may protect against depolymerization of α-tubulin by sequestering the proteolytic cleavage sites from calpain

Finally, HSPBs may prevent cardiomyocyte remodeling via inhibition of proteases, such as calpain. In tachypaced *Drosophila* with dmHSP23 overexpression, likely the functional ortholog of human HSPB1 prevented the activation of calpain and myolysis and heart wall contractile dysfunction (Zhang et al. 2011). This finding is in line with a study showing that HSPB1 prevents ischemia/reperfusion-induced degradation of the contractile proteins cardiac troponin I and troponin T by interacting with the COOH-terminus and NH_2_-terminus, respectively. This interaction prevented calpain from cleaving cardiac troponin I and T and resulted in conservation of the contractile function in ventricular cardiomyocytes (Lu et al. 2008). Also, HSPB1 co-localizes with cardiac troponin T in ventricular cardiomyocytes after morphine withdrawal, thereby preventing its degradation by calpain and maintaining myocardial function (Martinez-Laorden et al. 2015). These findings together imply that HSPB1 binds to contractile proteins, thereby sequestering the proteolytic cleavage regions from calpain (Fig. 1).

### HSPB in patients with AF

The experimental findings on the protective role of HSPB members in AF are in line with observations in paroxysmal and persistent AF patients. In patients with AF, an inverse correlation between the amount of HSPB1 expression and the level of myolysis and the duration of persistent AF is found (Brundel et al. 2006a). Patients with long-standing persistent AF reveal reduced levels of HSPB1 compared to persistent AF patients, suggesting that HSPB1 induction may represent a therapeutic target in long-standing persistent AF patients. Furthermore, HSPB members are found to represent a biomarker for AF onset and progression and may also predict the clinical outcome after interventions. A recent study showed that the serum HSPB1 levels of patients who received catheter ablation predict AF recurrences. Patients with high levels of HSPB1 in serum show improved maintenance rate of sinus rhythm (Hu et al. 2012). Because of the pleiotropic cardioprotective effects of HSPB1 on AF substrate formation, HSP inducers currently represent a class of drugs with promising therapeutic potential in clinical AF.

## Therapeutic application of HSP induction in experimental and clinical AF

Previous research has demonstrated that the (genetic) induction of HSPB members provides prevention effect on tachycardia-induced structural remodeling and contractile dysfunction. A drug often used to boost HSP expression is geranylgeranylacetone (GGA) (Hoogstra-Berends et al. 2012). GGA is originally used as an anti-ulcer agent and is a non-toxic acyclic isoprenoid compound with a retinoid skeleton that induces HSP synthesis in various tissues, including gastric mucosa, intestine, liver, heart, retina, and the central nervous system (Katsuno et al. 2005; Ooie et al. 2001). GGA induces HSP expression probably via the activation of the heat shock transcription factor 1 (Ke et al. 2008). The protective effect of GGA-induced HSP expression on structural remodeling has been observed in experimental models of AF, suggesting that the induction of HSPs by GGA may have a potential value for clinical AF (Brundel et al. 2006a; Ke et al. 2008). In tachypaced *Drosophila*, GGA treatment protects against contractile dysfunction of the heart wall and structural remodeling (Zhang et al. 2011). Furthermore, in canine models for (acute) atrial ischemia-related AF and tachypacing-induced AF promotion, GGA treatment reveals protective effects against cardiomyocyte remodeling and consequently occurrence and recurrence of AF after cardioversion (Brundel et al. 2006a; Sakabe et al. 2008).

In addition to the pharmacological induction of HSPB, exercise is also found to induce HSPB levels and subsequently reveal cardioprotective effects. Various studies show that gene and protein levels of HSPB1 and HSPB6 are elevated after physical exercise in rat and mouse models. Interestingly, in these studies, HSPB1 and HSPB6 were phosphorylated, resulting in stabilization of myofilaments, restoration of disrupted contractile proteins, and consequently improved contractile function of the heart (Boluyt et al. 2006; Burniston 2009; Campos et al. 2012; de Moraes et al. 2015; Rinaldi et al. 2006; Sakamoto et al. 2006). Therefore, physical exercise may represent a promising therapeutic therapy to ameliorate the cardiac function and quality of life in patients with AF and maintain normal sinus rhythm after cardioversion, via induction of HSPB levels.

## Conclusion

Various HSPB members conserve a healthy proteostasis of cardiomyocytes and thereby prevent AF onset and progression. Their mode of action is via the stabilization of the cardiomyocyte structure, thus conserving the contractile and electrophysiological function of the atria. Since compounds, such as GGA, and exercise are found to induce HSPB expression, these may represent promising novel therapeutic strategies to prevent AF onset and progression.
